# A Cadaveric Study of the Martin-Gruber Anastomosis Morphology

**DOI:** 10.7759/cureus.78139

**Published:** 2025-01-28

**Authors:** Paul Tran, Dallas Bennett, Mathew Mendoza, Albert Sarpong, Madeline Ayala, Chakravarthy Sadacharan, Samantha P Tippen

**Affiliations:** 1 Department of Biomedical Sciences, Tilman J. Fertitta Family College of Medicine, Houston, USA; 2 Department of Anatomy, Baylor College of Medicine, Houston, USA

**Keywords:** flexor digitorum profundus (fdp), martin-gruber anastomosis (mga), median nerve (mn), peripheral nerve variations, ulnar nerve (un)

## Abstract

Introduction and aim: The Martin-Gruber anastomosis (MGA) is a neural communication between the median nerve (MN) and ulnar nerve (UN), typically located in the proximal forearm. Despite its clinical significance in diagnosing and treating nerve pathologies such as carpal and cubital tunnel syndromes, anatomical variations of MGA remain underexplored. This cadaveric study aimed to determine MGA prevalence, characterize its morphological patterns, and evaluate implications for peripheral nerve surgeries such as ulnar nerve transplantation and upper extremity neuropathies.

Methods: Cadaveric dissections of 101 forearms were conducted at Tilman J. Fertitta Family College of Medicine and Baylor College of Medicine. Only intact MGA specimens were included. Nerves were exposed by reflecting fascia and muscle layers, and MGA pathways were traced retrogradely. Measurements included the distance of MGA origins from the medial epicondyle (ME) and the width of the communication ramus. A custom classification system categorized MGA patterns based on anatomical configurations.

The Martin-Gruber anastomosis prevalence found within the study was 37.6% (n = 38/101), with type I being the most common at 58% (n = 22/38), followed by type III at 32% (n = 12/38), and type II at 11% (n = 4/38). Unilateral MGA predominated at 53% (n = 20/38) and most patterns were intramuscularly embedded in the flexor digitorum profundus (FDP) muscle. Communication fiber lengths ranged from 3 to 10 cm from the ME. Findings corroborate prior classifications while highlighting subtle variations in morphology.

Conclusion: This study reveals a higher MGA prevalence compared to global averages, underscoring its clinical importance. Detailed morphological insights enhance understanding of MGA variations, aiding surgical precision, and improving outcomes in nerve repair and transplantation. Further research on MGA functional dynamics is recommended to refine classification frameworks and surgical approaches.

## Introduction

The median nerve (MN) is formed by the convergence of the medial (C8, T1) and lateral (C5, C6, C7) cords of the brachial plexus [1]. In contrast, the ulnar nerve (UN) arises as a terminal branch of the medial cord [1]. While both nerves are essential for sensory and motor functions in the upper extremities, their intricate anatomical variations remain underexplored.

The Martin-Gruber anastomosis (MGA) is a significant neural communication between the MN and UN in the proximal-to-mid forearm [2]. First proposed by Martin, it was described as a potential nerve communication ramus between the two nerves [3]. Gruber later confirmed its existence through a dissection study, reporting a prevalence of 15.2% [4]. A meta-analysis by Roy et al., based on 41 studies, estimated the global prevalence at 19.5% and noted the highest prevalence in North Americans (29.1%) [1,5]. Genetic studies have suggested that MGA may be linked to congenital abnormalities and familial dominant inheritance patterns [6,7].

MGA has clinical significance in upper extremity nerve injuries and conditions such as carpal tunnel syndrome and cubital tunnel syndrome [8]. Additionally, understanding the anatomical parameters of MGA is crucial for improving the success of peripheral nerve surgeries, including nerve transposition procedures [8,9].

Despite numerous studies on its prevalence, patterns, and functions, findings vary significantly [10-13]. This study aimed to enhance knowledge of MGA prevalence and patterns through cadaveric dissections and detailed nerve measurements to aid surgical and clinical applications.

## Materials and methods

A total of 101 human cadaveric forearms were dissected at Tilman J. Fertitta Family College of Medicine and Baylor College of Medicine to investigate MGA pathways. Forearms with visually intact and undamaged muscle tissue were included in the study. Forearms with trauma, injury, or unidentifiable MGA were excluded. Cadaveric specimens were stored at optimal temperatures and within appropriate storage units to ensure proper tissue preservation prior to dissections.

The dissections involved removing fascia and muscle layers to expose the MN and UN from the distal flexor retinaculum to the proximal cubital fossa. The forearms were carefully dissected until both fascia and muscle layers were present. Both the MN and UN were identified and then traced from the distal flexor retinaculum to the proximal cubital fossa. Any overlapping muscles were reflected during this process. A series of vertical incisions and blunt dissections were performed to expose underlying MGA nerves and branches. MGA was then confirmed by tracing retrogradely along the MN and UN for communicating connections.

Patterns were documented, photographed, and compared to existing studies to determine prevalence and morphology. Measurements via digital caliper were taken relative to the medial epicondyle (ME) and included - ME to the origin of the MGA from the UN, ME to the origin of the MGA from the MN, and width of the MGA communication ramus between the MN and UN.

## Results

This study found an MGA prevalence of 37.6% (n = 38/101). Type I anastomosis had the highest prevalence at 58% (n = 22/38), followed by type III at 32% (n = 12/38) and type II at 11% (n = 4/38). Unilateral MGA was more common (53%, n = 20/38) compared to bilateral distribution (47%, n = 18/38).

**Figure 1:**
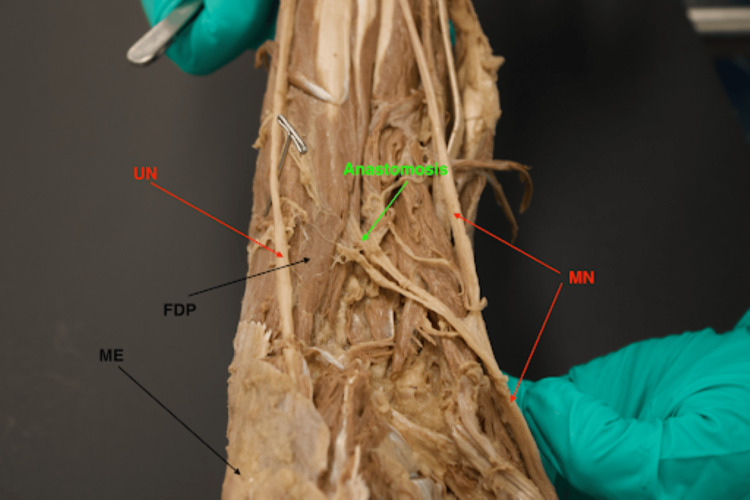
Cadaveric forearm dissection showcases MGA type I pattern. The arrows within this figure highlight key landmarks involved in identifying MGA pattern type I. MGA is found between both the MN and UN. ME is used as a directional reference indicating the orientation of the forearm (distal toward north of the image). Note that the MN was retracted laterally to better depict the route of MGA. MGA: Martin-Gruber anastomosis; MN: median nerve; ME: medial epicondyle; FDP: flexor digitorum profundus; UN: ulnar nerve

**Figure 2:**
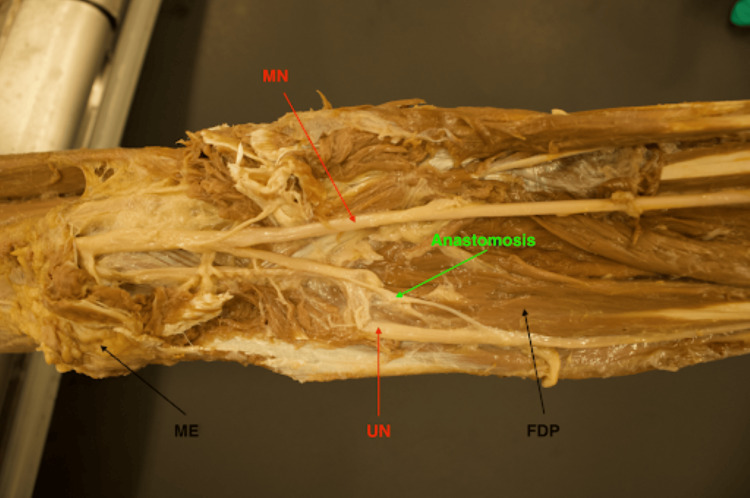
Cadaveric forearm dissection showcases MGA type II pattern. The arrows within this figure highlight key landmarks that are involved in identifying MGA pattern type II. MGA is found between the MN and UN. ME is used as a directional reference indicating the orientation of the forearm (distal toward right of the image). Type II anastomosis can be seen here lying superficial to the FDP muscle. MGA: Martin-Gruber anastomosis; MN: median nerve; ME: medial epicondyle; FDP: flexor digitorum profundus; UN: ulnar nerve

**Figure 3:**
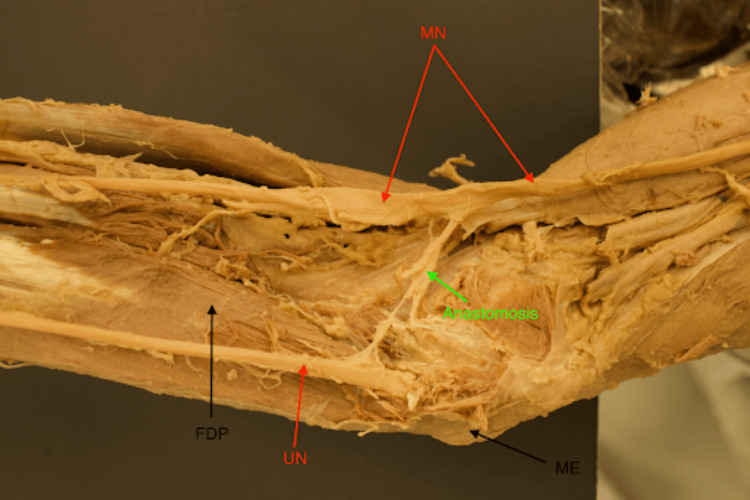
Cadaveric forearm dissection showcases MGA type III pattern. The arrows within this figure highlight key landmarks that are involved in identifying MGA pattern type III. MGA is found between the MN and UN. ME is used as a directional reference indicating the orientation of the forearm (distal toward left of the image). Note that the anastomosis is found in closer proximity to the ME compared to other patterns and more adjacent to the cubital fossa. MGA: Martin-Gruber anastomosis; MN: median nerve; ME: medial epicondyle; FDP: flexor digitorum profundus; UN: ulnar nerve

The MGA was frequently embedded within the fibers of the flexor digitorum profundus (FDP) muscle. Some studies have used the ulnar artery as a landmark for MGA pattern classification [14]. To account for observed variations, a custom classification system was developed for this study. The patterns were categorized as follows: type I - MGA embedded deep within the FDP muscle (Figure 1); type II - MGA located near or superficial to the FDP muscle (Figure 2); and type III - MGA situated proximally near the cubital fossa, adjacent to or within the FDP muscle window (Figure 3).

**Figure 4:**
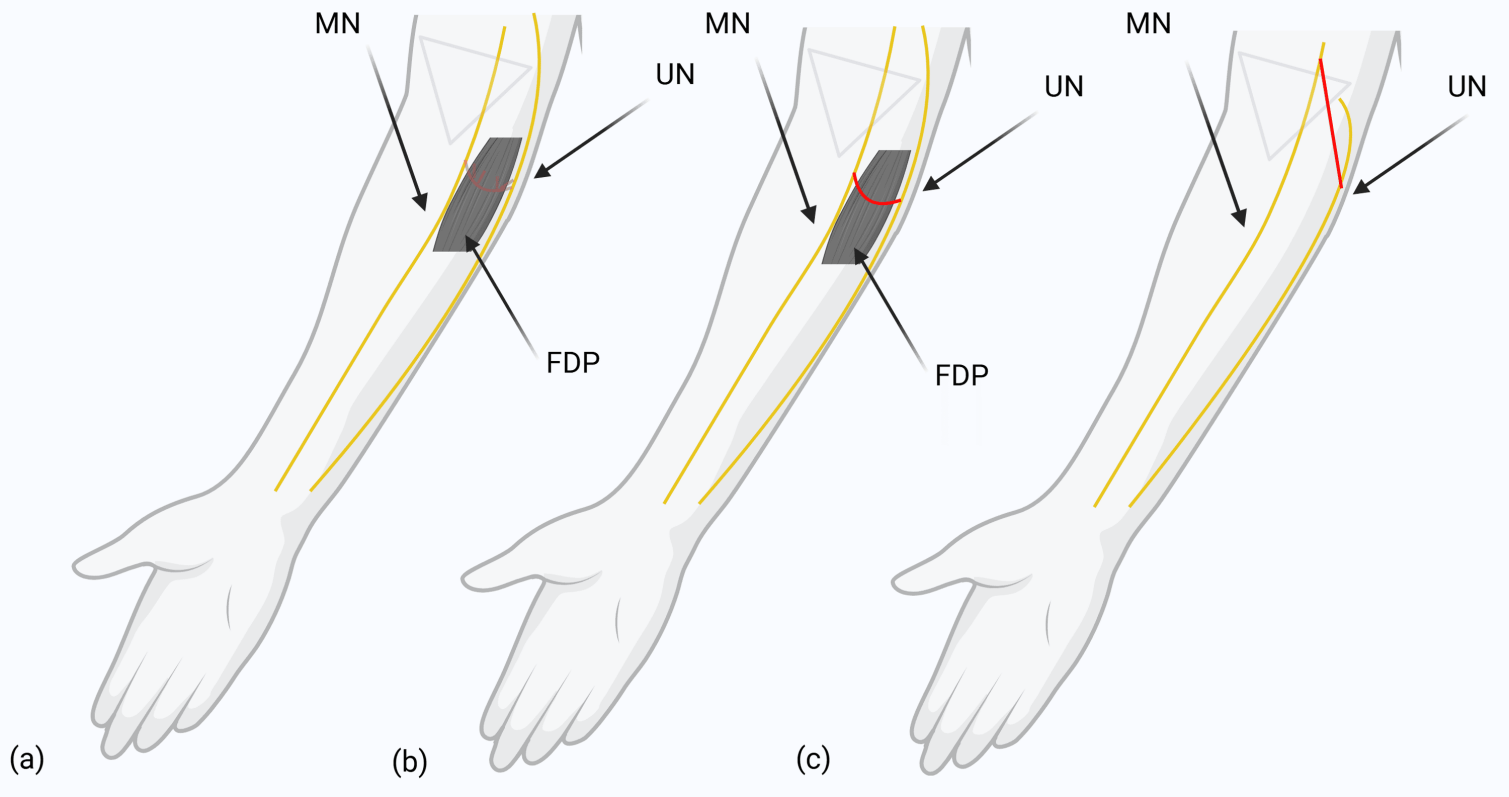
Diagram depicting MGA pattern types. The arrows within this figure highlight key landmarks that indicate characteristics to distinguish between different MGA patterns. The anastomosis connection (shown in red) is found between the MN and UN. FDP muscle is an important reference muscle that denotes key differences between pattern types. The image is created by the author (Paul Tran) of this study using BioRender.com. MGA: Martin-Gruber anastomosis; MN: median nerve; ME: medial epicondyle; FDP: flexor digitorum profundus; UN: ulnar nerve

All MGA patterns originated from the MN and extended distally to the UN. These patterns were further illustrated with reference diagrams to provide a clear visual representation (Figures 4a-4c). Note that MGA pattern types are depicted in ascending alphabetical order. Parameter MGA measurements of averages can be seen illustrated in Table 1.

**Table 1:** A list of measurements in reference to the three lengths assessed for Martin-Gruber anastomosis parameters. MN: median nerve; ME: medial epicondyle; UN: ulnar nerve

Measurement	Measurement estimated length averages
ME to UN	7.8 ± 3.4 cm
ME to MN	6.1 ± 2.2 cm
Width (MN to UN)	5.7 ± 2.1 cm

## Discussion

The Martin-Gruber anastomosis (MGA) is a clinically significant anatomical variation with direct implications for diagnosing and treating peripheral nerve pathologies. A chronological reference table highlights various related studies that have reported MGA prevalence (Table 2) [4,12,13,15-20]. Situated distal to the cubital fossa, the MGA frequently intermingles with the flexor digitorum profundus (FDP) muscle, highlighting its importance in both surgical interventions and understanding upper extremity nerve injuries [8,9,12,13,21]. Previous studies have correlated MGA with paradoxical sensory and motor deficits, which can complicate the diagnosis of median nerve (MN) and ulnar nerve (UN) lesions. Notably, conditions like carpal tunnel syndrome and cubital tunnel syndrome may be misdiagnosed without a comprehensive understanding of MGA patterns and their functional implications [9,12,13].

**Table 2:** Summary of reported studies regarding Martin-Gruber anastomosis prevalence including year, author, and study logistics.

Studies	Year	Number of cases	Study type	Martin-Gruber anastomosis prevalence (%)
Gruber [4]	1870	250	Dissection	15.2
Thomson [15]	1893	406	Dissection	15.5
Kimura et al. [16]	1976	328	Electrophysiology	17
Amoiridis [17]	1992	100	Electrophysiology	32
Nakashima [18]	1993	108	Dissection	21.3
Shu et al. [19]	1999	72	Dissection	23.6
Lee et al. [20]	2005	102	Dissection	39.2
Cavalheiro et al. [12]	2016	100	Dissection	27
Bharathi et al. [13]	2023	60	Dissection	18.3
Present Study	2024	101	Dissection	37.6

This study reports a prevalence of 37.6% (n = 38) for MGA, higher than the global median prevalence of 19.5% (n = 10,562) as documented in prior meta-analyses [1,10]. Like findings by Lee et al., a significant proportion of MGA patterns observed in this study were intramuscular, embedded within the FDP, consistent with their type III classification [20]. Additionally, the study aligns with the findings of Cavalheiro et al., which reported high frequencies of intramuscular communications and complex branching patterns, emphasizing the diverse morphological presentations of MGA [12].

The observed MGA patterns in this study predominantly correspond to previously documented classifications, with the most common resembling type I configurations involving communication between branches of the MN and UN within or adjacent to the FDP muscle. These findings are consistent with Nakashima and Shu et al., who highlighted similar intramuscular anastomoses [18,19]. However, subtle variations in branching and insertion sites underline the need for continued investigation into these configurations to refine existing classifications.

The length of the MGA communication fibers, ranging from 3 cm to 10 cm from the medial epicondyle (ME), also aligns with earlier studies. For instance, Bharathi et al. and other anatomical studies have reported comparable ranges, with variations attributable to population-based anatomical differences and dissection techniques [13,22]. These measurements are crucial for guiding surgical approaches, particularly during nerve transposition or repairs, where accurate identification of MGA is essential to minimize iatrogenic injuries.

Historically, numerous classification systems have been proposed, each with unique contributions to understanding MGA variations. For instance, Bharathi et al. outlined three types of MGA configurations, while Cavalheiro et al. expanded this framework to six distinct patterns, incorporating both intramuscular and extra-muscular communications [12,13]. Similarly, earlier studies by Shu et al. and Lee et al. further characterized MGA patterns based on connections between the anterior interosseous nerve (AIN), MN, and UN [19,20]. These diverse classifications underscore the complexity of MGA and the need for standardized frameworks to facilitate cross-study comparisons.

The findings of this study also emphasize the unilateral predominance of MGA, a result consistent with global trends reported by Amoiridis and others [17]. The anatomical variability observed here highlights the importance of thorough knowledge of MGA configurations to optimize clinical outcomes in nerve repair surgeries and to explain unexpected functional outcomes in patients presenting with peripheral neuropathies. 

While the study provides valuable insights into MGA prevalence and morphology, there are also several limitations to take into consideration. The occasional inability to definitively confirm some MGA-like patterns as true anastomoses indicate an area for further exploration. In addition, due to the delicate and fibrous nature of MGA connections, underlying nerve pathways were prone to a relatively high risk for accidental transaction or removal prior to identification. Future research employing advanced imaging modalities or neurophysiological studies may better elucidate these connections and their implications for surgical and rehabilitative practices.

## Conclusions

This study highlights the higher prevalence of MGA (37.6%, n = 38) compared to global averages, emphasizing its clinical and surgical relevance for planning and post-operational outcomes. The findings also reinforce previous observations of MGA intramuscular configurations within the FDP muscle and unilateral predominance. Despite its alignment with existing literature, subtle pattern variations suggest that further investigations into MGA's functional and anatomical complexities are warranted, particularly for refining nerve transplantation and repair strategies.
